# Reductions in carotid chemoreceptor activity with low‐dose dopamine improves baroreflex control of heart rate during hypoxia in humans

**DOI:** 10.14814/phy2.12859

**Published:** 2016-07-14

**Authors:** Michael T. Mozer, Walter W. Holbein, Michael J. Joyner, Timothy B. Curry, Jacqueline K. Limberg

**Affiliations:** ^1^ Department of AnesthesiologyMayo ClinicRochesterMinnesota

**Keywords:** Baroreflex, blood pressure, chemoreflex, dopamine, heart rate, tidal volume

## Abstract

The purpose of the present investigation was to examine the contribution of the carotid body chemoreceptors to changes in baroreflex control of heart rate with exposure to hypoxia. We hypothesized spontaneous cardiac baroreflex sensitivity (scBRS) would be reduced with hypoxia and this effect would be blunted when carotid chemoreceptor activity was reduced with low‐dose dopamine. Fifteen healthy adults (11 M/4 F) completed two visits randomized to intravenous dopamine or placebo (saline). On each visit, subjects were exposed to 5‐min normoxia (~99% S_p_O_2_), followed by 5‐min hypoxia (~84% S_p_O_2_). Blood pressure (intra‐arterial catheter) and heart rate (ECG) were measured continuously and scBRS was assessed by spectrum and sequence methodologies. scBRS was reduced with hypoxia (*P* < 0.01). Using the spectrum analysis approach, the fall in scBRS with hypoxia was attenuated with infusion of low‐dose dopamine (*P* < 0.01). The decrease in baroreflex sensitivity to rising pressures (scBRS “up‐up”) was also attenuated with low‐dose dopamine (*P* < 0.05). However, dopamine did not attenuate the decrease in baroreflex sensitivity to falling pressures (scBRS “down‐down”; *P* > 0.05). Present findings are consistent with a reduction in scBRS with systemic hypoxia. Furthermore, we show this effect is partially mediated by the carotid body chemoreceptors, given the fall in scBRS is attenuated when activity of the chemoreceptors is reduced with low‐dose dopamine. However, the improvement in scBRS with dopamine appears to be specific to rising blood pressures. These results may have important implications for impairments in baroreflex function common in disease states of acute and/or chronic hypoxemia, as well as the experimental use of dopamine to assess such changes.

## Introduction

The carotid body chemoreceptors are located bilaterally at the bifurcation of the common carotid artery and are responsible for monitoring the partial pressure of oxygen in arterial blood (Kumar and Bin‐Jaliah 2007; Kumar and Prabhakar 2012). Activation of the carotid body chemoreceptors via decreases in the partial pressure of oxygen (hypoxemia) initiates reflex increases in minute ventilation and sympathetic nervous system activity (Kumar and Bin‐Jaliah 2007; Kumar 2009). Hypoxemia is a common occurrence for patients with chronic cardiorespiratory illness (e.g., heart failure, chronic obstructive pulmonary disorder, sleep apnea, among others) (Kent et al. 2011; Oldenburg et al. 2016). Chronic activation of the carotid chemoreceptors in clinical conditions such as heart failure (Ponikowski et al. 2001) has been linked to increased cardiovascular morbidity and mortality. Part of this effect may be related to increases in carotid chemoreceptor sensitivity and subsequent effects on baroreflex sensitivity (Ponikowski et al. 1999; Del Rio et al. 2013; Niewinski et al. 2013).

Located within the carotid sinus, the arterial baroreceptors play an important role in blood pressure regulation (Cowley et al. 1973). Maintaining sensitivity of the arterial baroreceptors is integral to preventing large fluctuations in blood pressure which can result in end‐organ damage. There are known interactions between the carotid chemoreceptors and baroreceptors, such that the normal heart rate response to hypoxia is attenuated during baroreceptor loading (Somers et al. 1991). Additionally, acute hypoxia has been shown to blunt cardiac baroreflex sensitivity (Heistad et al. 1971; Sagawa et al. 1997; Roche et al. 2002; Steinback et al. 2009); thus, repeated hypoxic exposures could contribute to impairments in baroreflex sensitivity and increases in cardiovascular disease risk.

Previous human models for investigating the contribution of the carotid chemoreceptors to autonomic hemodynamic regulation have relied on hyperoxia to blunt chemoreceptor activity (Ponikowski et al. 1999; Hering et al. 2007; Limberg et al. 2014; Sinski et al. 2014; Edgell et al. 2015). However, the experimental model of hypoxic exposure limits the use of hyperoxia as a means of concurrent carotid chemoreceptor desensitization. In contrast, intravenous infusion of dopamine in low doses has been used in many studies in humans to acutely depress peripheral chemosensitivity to hypoxia (Welsh et al. 1978; Bainbridge and Heistad 1980; Ward and Bellville 1982, 1983; Boetger and Ward 1986; Sabol and Ward 1987; Bascom et al. 1991; Henson et al. 1992; Dahan et al. 1996; Ward et al. 2009; Stickland et al. 2011). With this information in mind, we sought to examine the contribution of carotid chemoreceptors to baroreflex control of heart rate during hypoxia in healthy adults. We hypothesized spontaneous cardiac baroreflex sensitivity would be reduced from baseline during hypoxia, and that this reduction would be attenuated with infusion of dopamine.

## Methods

### Institutional approval and informed consent

Written informed consent was obtained from all subjects. All procedures were approved by the Institutional Review Board at the Mayo Clinic and conformed to the standards set by the Declaration of Helsinki.

### Participants

Fifteen participants (11 male/4 female) completed a dopamine dose–response visit and two study visits randomized to intravenous infusion of dopamine or placebo (saline). All participants (22–42 years of age) were healthy, nonobese (BMI ≤ 30 kg · m^−2^), nonsmokers without chronic diseases, and taking no medications known to affect cardiovascular, respiratory, or autonomic function. Women were not pregnant (confirmed by negative pregnancy test prior to participation) and were studied in the placebo phase of oral contraceptive use (*n* = 4 on hormonal contraceptive). Subjects refrained from alcohol, caffeine, and exercise for 24 h and fasted for 12 h prior to the dose–response visit and the study visits.

#### Dopamine dose–response visit

Data collected from the dopamine dose–response visit were published previously (Limberg et al. 2016). During the visit, subjects were rested in a semisupine position and over the course of 2.5 h, completed five trials to determine their ventilatory response to hypoxia during four different doses of dopamine. An intravenous catheter was placed for infusion of dopamine and breath‐by‐breath ventilation and inspired/expired gasses were monitored using a free‐standing metabolic cart (Ultima CardiO_2_; MCG Diagnostics, Saint Paul, MN). Subjects breathed through a two‐way non‐rebreathing valve connected to a switching valve. Each trial consisted of 15 min of room air breathing followed by hypoxic response testing (Limberg et al. 2016). Briefly, each hypoxic response test began with 2–6 breaths of nitrogen, followed by room air for 2 min (Niewinski et al. 2014). This was repeated four times during each dopamine infusion trial, achieving oxygen saturation levels of 70–99% (as assessed by pulse oximetry). After completion of the final nitrogen condition, the next trial began. Trials were completed in the following order: (1) Saline, (2) Dopamine 1 *μ*g · kg^−1 ^· min^−1^, (3) 2 *μ*g · kg^−1^·min^−1^, (4) 3 *μ*g·kg^−1^·min^−1^, (5) 4 *μ*g·kg^−1^·min^−1^. The hypoxic ventilatory response was calculated as follows: (1) for each administration of 100% nitrogen, the three largest consecutive breaths were averaged; (2) simultaneous oxygen saturation (%S_p_O_2_) was collected and the nadir was recorded for each nitrogen administration, (3) chemosensitivity to hypoxia was assessed as the slope of the linear regression line for average ventilation (L·min^−1^) and nadir oxygen saturation (%S_p_O_2_) from each nitrogen breathing trial (Niewinski et al. 2014). The individualized dose of dopamine (1, 2, 3, or 4 *μ*g·kg^−1^·min^−1^) that resulted in a maximum reduction in the hypoxic ventilatory response with minimal effect on cardiovascular measures was used for the subsequent study visit (average dose: 2.3 ± 0.3 *μ*g·kg^−1^·min^−1^). See Limberg et al. (2016) for additional information.

#### Baroreflex study visits

A minimum of 1 week following completion of the dopamine dose–response visit, subjects participated in two study visits, each separated by a minimum of 1 week and randomized to intravenous dopamine (individualized dose of dopamine that resulted in a maximum reduction in the hypoxic ventilatory response) or placebo (saline). Subjects and research personnel were blinded to condition. Only pharmacy and nursing staff were aware of study condition until each subject completed both visits, at which time, all other research personnel were unblinded.

### Monitoring

An intravenous catheter was inserted in the dominant arm for dopamine/placebo infusion. A brachial arterial catheter (20 gauge, 5 cm) was inserted in the nondominant arm using aseptic techniques under local anesthesia (2% lidocaine) to measure beat‐to‐beat blood pressure (TruWave Pressure Transducer; Edwards Lifescience, Irvine, CA). Heart rate was monitored with a three‐lead electrocardiogram (Cardiocap/5; Datex‐Ohmeda Inc, Louisville, CO) and arterial oxygen saturation by pulse oximetry (Masimo Corporation, Irvine, CA). Subjects breathed on a mouthpiece/mask connected to a two‐way, non‐rebreathing valve. Breath‐by‐breath tidal volumes (Universal Ventilation Meter; VacuMed, Ventura, CA), respiratory rate, and inspired/expired gasses (Cardiocap/5; Datex‐Ohmeda Inc, Louisville, CO) were assessed and inspiratory time was calculated.

### Experimental protocol

Subjects rested supine. Following a 10‐min infusion (Saline/Dopamine) period, subjects were exposed to 5‐min normoxia (medical air, 21% oxygen; Saline 300 ± 1 sec, Dopamine 298 ± 1 sec, *P* > 0.05) followed by ~5‐min hypoxia (Saline 261 ± 19 sec, Dopamine 286 ± 9 sec, *P* > 0.05). Hypoxic conditions were poikilocapnic and hypoxemia was achieved by titrating inspired oxygen levels using a gas blender (Inspired oxygen: Saline 13.9 ± 0.3%, Dopamine 15.1 ± 0.4%) to achieve an oxygen saturation of ~85% (S_p_O_2_: Saline: 84 ± 1%, Dopamine: 84 ± 1%). Arterial blood gasses were confirmed in a subset of subjects (*n* = 6; S_p_O_2_: Saline: 84 ± 1%, Dopamine: 85 ± 1%; S_a_O_2_: Saline: 84 ± 1%, Dopamine: 82 ± 2%). The average transition phase from room air to the desired S_p_O_2_ was ~4 min (Saline 263 ± 11 sec, Dopamine 221 ± 10 sec) and the 5‐min time period for hypoxia began when ~85% S_p_O_2_ was achieved.

### Data and statistical analysis

All hemodynamic measurements were collected digitally using a PowerLab data acquisition system (ADinstruments, Inc., Colorado Springs, CO) with a sampling rate of 1000 Hz. To determine baroreflex sensitivity, a standard spectrum analysis method was used (WinCPRS, Version 1.163; Absolute Aliens Oy, Turku, Finland). Normal oscillations in heart rate and blood pressure were manipulated via fast Fourier transform to divide variability into frequency components. Cross‐spectrum analysis was used to compare variability in frequency between the R‐R interval and systolic blood pressure spectra. Spontaneous cardiac baroreflex sensitivity (scBRS) was defined as the mean gain of the low‐frequency (0.04–0.15 Hz) transfer function of the resulting cross spectrum after phase correction with a strong spectra‐to‐spectra correlation (*R* > 0.50) (Mulder and Mulder 1981; Robbe et al. 1987; Wichterle et al. 2000a,b; Pinna 2007; van de Vooren et al. 2007). scBRS was also assessed from the same sections of data using the sequence method (WinCPRS, Version 1.163; Absolute Aliens Oy) (Parati et al. 1988). The sequences used for systolic blood pressure and R‐R interval signals were required to rise or fall monotonically in the same direction for at least three consecutive beats. Changes in systolic blood pressure and R‐R interval values were required to exceed 1 mmHg and 5 ms, respectively, and analysis was performed separately for ascending (up‐up) and descending (down‐down) sequences. Those individuals where no sequences could be identified during any ~5‐min period (*n* = 2) were excluded from the analysis. Values belonging to the identified sequences were formed into xy‐pairs and a regression curve was fitted, with the slope of the curve equaling baroreflex sensitivity (ms·mmHg^−1^). The absolute (Δ, Hypoxia‐Normoxia) and relative (% [Hypoxia‐Normoxia]/Normoxia × 100]) changes in scBRS with hypoxia were compared between saline and dopamine conditions.

Statistical analysis was completed using SigmaStat 12.0 software (Systat Software Inc., San Jose, CA). The primary analysis was to examine the effect of hypoxia on baroreflex sensitivity under saline and dopamine conditions. A one‐way repeated measures analysis of variance was used to examine the effect of hypoxia on main outcome variables. In all cases, distributional assumptions were assessed and nonparametric tests (Friedman repeated measures analysis of variance on ranks) were used when necessary. One‐way repeated measures analysis of variance was used to examine the differences in the effect of saline/dopamine on change (absolute [Δ], relative [%]) variables. Further comparisons using Pearson correlations were conducted post hoc to determine the potential contribution of secondary outcome variables (e.g., heart rate, tidal volume, respiratory rate, inspiratory time) on changes in baroreflex sensitivity. An alpha of *P* < 0.05 was considered statistically significant and data were expressed as mean ± standard error of the mean.

## Results

**Figure 1:**
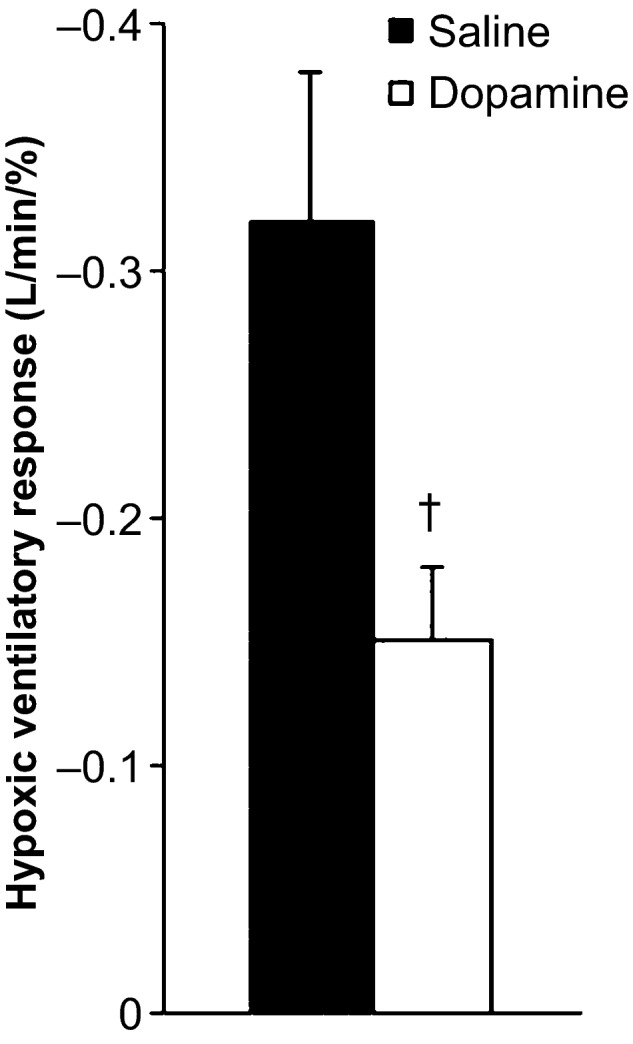
Effect of low‐dose dopamine on the hypoxic ventilatory response. ^†^ *P* < 0.05 versus saline.

Subject demographics are provided in Table 1. All subjects (11 M/4 F) were relatively young (31 ± 1 years), nonobese (BMI 25 ± 1 kg·m^−2^), and not taking medications. When assessed during the dopamine dose–response visit, the ventilatory response to acute hypoxia (a measure of carotid body chemoreflex sensitivity) was significantly reduced with low‐dose dopamine (−0.32 ± 0.06 to −0.15 ± 0.03 L·min^−1^·%^−1^, *P* < 0.05; Fig. 1) at the selected individualized dose (average dose: 2.3 ± 0.3 *μ*g·kg^−1^·min^−1^).

**Table 1:** Subject demographics

Sex	11 M/4 F
Age (years)	31 ± 1
Height (cm)	178 ± 3
Weight (kg)	80 ± 5
BMI (kg·m^−2^)	25 ± 1
HVR (L·min^−1^·%^−1^)	−0.32 ± 0.06

Data are reported as mean ± SEM. BMI, body mass index; HVR, hypoxic ventilatory response; M, male; F, female.

Steady‐state hemodynamic responses are reported in Table 2. Hypoxia resulted in significant increases in heart rate (*P* < 0.01) that were not different between saline and dopamine conditions (Δ, *P* = 0.29; %, *P* = 0.09). Changes in blood pressure (systolic, diastolic, mean) were neither observed with hypoxia (*P* > 0.05) nor low‐dose dopamine infusion (*P* > 0.05).

**Table 2:** Steady‐state hemodynamic responses

	Normoxia	Hypoxia	Δ	Delta (%)
Heart rate (beats·min^−1^)				
Saline	62 ± 2	80 ± 3^a^	19 ± 3	31 ± 5
Dopamine	66 ± 2	83 ± 3^a^	16 ± 3	25 ± 4
Systolic blood pressure (mmHg)				
Saline	143 ± 2	143 ± 5	0 ± 3	0 ± 2
Dopamine	142 ± 3	147 ± 4	5 ± 2	3 ± 2
Diastolic blood pressure (mmHg)				
Saline	76 ± 1	77 ± 2	0 ± 1	0 ± 1
Dopamine	73 ± 1	75 ± 1	1 ± 1	2 ± 1
Mean blood pressure (mmHg)				
Saline	99 ± 2	99 ± 3	0 ± 1	0 ± 1
Dopamine	96 ± 1	98 ± 2	2 ± 1	2 ± 1

Data are reported as mean ± SEM from *n* = 15.

a *P* < 0.05 versus Normoxia, *P* < 0.05 versus Saline.

Steady‐state ventilatory responses are reported in Table 3. As designed, there was a significant reduction in inspired oxygen during hypoxic conditions (*P* < 0.01) which resulted in significant reductions in oxygen saturation (*P* < 0.01). The inspired oxygen level necessary to achieve the same reduction in oxygen saturation was higher under dopamine conditions (15.1 ± 0.4%) when compared to saline conditions (13.9 ± 0.3%, *P* < 0.01). Steady‐state hypoxia resulted in significant increases in tidal volume (*P* < 0.01) and minute ventilation (*P* < 0.01), with no change in respiratory rate (*P* > 0.05). These responses were not significantly different between saline and dopamine conditions (range: *P* = 0.11–0.85). The rise in minute ventilation contributed to a slight fall (−0.5 ± 0.1%) in end‐tidal CO_2_ (*P* < 0.01) that was not different between saline and dopamine conditions (Δ: *P* = 0.78; %: *P* = 0.37). Inspiratory time was significantly increased with hypoxia, but only under saline conditions (Saline: *P* = 0.03; Dopamine: *P* = 0.16).

**Table 3:** Steady‐state ventilatory responses

	Normoxia	Hypoxia	Δ	Delta (%)
Respiratory rate (breaths·min^−1^)				
Saline	14 ± 1	14 ± 1	0 ± 0	−1 ± 2
Dopamine	13 ± 1	14 ± 1	1 ± 1	7 ± 4
Tidal volume (mL)				
Saline	559 ± 62	814 ± 61*	256 ± 27	54 ± 8
Dopamine	530 ± 42	727 ± 54*	197 ± 30	40 ± 6
Minute ventilation (L·min^−1^)				
Saline	7.8 ± 0.8	11.3 ± 0.8*	3.4 ± 0.4	51 ± 8
Dopamine	6.8 ± 0.4	10.0 ± 0.7*	3.2 ± 0.5	49 ± 8
Inspiratory time (sec)				
Saline	2.3 ± 0.2	2.1 ± 0.2*	−0.2 ± 0.1	−10 ± 5
Dopamine	2.6 ± 0.2	2.3 ± 0.3	−0.3 ± 0.2	−11 ± 7
End‐tidal CO_2_ (mmHg)				
Saline	41 ± 1	37 ± 1*	−4 ± 1	−10 ± 1
Dopamine	44 ± 1	40 ± 1*	−4 ± 1	−9 ± 1
Inspired O_2_ (%)				
Saline	21	13.9 ± 0.3*	−8.1 ± 0.3	−37 ± 1
Dopamine	21	15.1 ± 0.4*	−6.6 ± 0.3†	−30 ± 2†
S_p_O_2_ (%)				
Saline	99 ± 0	84 ± 1*	−14 ± 1	−14 ± 1
Dopamine	98 ± 0	84 ± 1*	−14 ± 1	−14 ± 1

Data are reported as mean ± SEM from *n* = 15 unless otherwise noted (inspiratory time, *n* = 13).

*P < 0.05 versus Normoxia, ^†^ *P* < 0.05 versus Saline.

**Figure 2:**
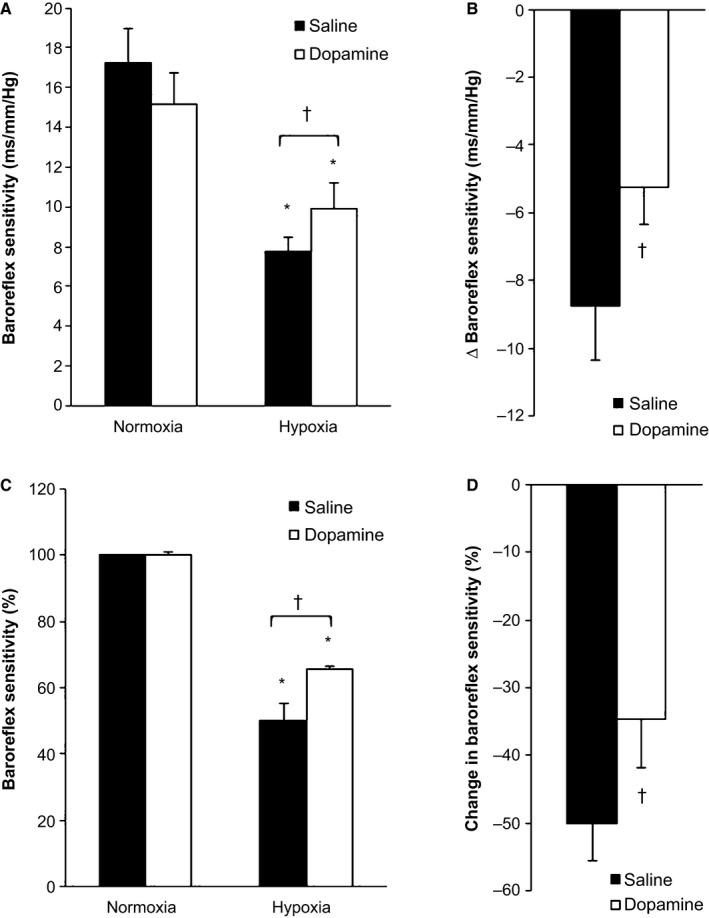
Spontaneous cardiac baroreflex sensitivity (spectrum analysis, low‐frequency transfer function). Data are reported as mean ± SEM from *n* = 15. *P < 0.05 versus Normoxia, ^†^ *P* < 0.05 versus Saline. Spontaneous cardiac baroreflex sensitivity (scBRS) was reduced during hypoxia when compared with normoxia (A: ms·mmHg^−1^, *P* < 0.01; C: %, *P* < 0.01). The decrease in scBRS with hypoxia during saline conditions was attenuated with low‐dose dopamine infusion (B: Δ, *P* < 0.01; D: %, *P* < 0.01).

Baroreflex sensitivity is reported in Table 4 and Figure 2. We found scBRS was reduced during hypoxia when compared with normoxia (*P* < 0.01), and this was observed using both spectrum and sequence methodologies. Using the spectrum analysis approach, the decrease in scBRS with hypoxia during saline conditions was attenuated with low‐dose dopamine infusion (Δ *P* < 0.01, % *P* < 0.01). The decrease in baroreflex sensitivity to rising blood pressures (Up‐Up Sequences) with hypoxia during saline was also attenuated with low‐dose dopamine (Δ *P* = 0.03, % *P* = 0.01). However, when the hypoxia‐mediated decrease in scBRS was assessed from Down‐Down Sequences, dopamine did not attenuate the fall (Δ *P* = 0.06, % *P* = 0.15).

**Table 4:** Spontaneous cardiac baroreflex sensitivity

	Normoxia	Hypoxia	Δ	Delta (%)
Baroreflex sensitivity LF transfer (ms·mmHg^−1^)				
Saline	17 ± 2	8 ± 1*	−9 ± 2	−50 ± 5
Dopamine	15 ± 2	10 ± 1*	−5 ± 1†	−35 ± 7†
Baroreflex sensitivity Up‐Up (ms·mmHg^−1^)				
Saline	20 ± 3	9 ± 1*	−11 ± 3	−43 ± 9
Dopamine	18 ± 2	11 ± 1*	−7 ± 2†	−28 ± 8†
Baroreflex sensitivity Down‐Down (ms·mmHg^−1^)				
Saline	21 ± 3	9 ± 1*	−12 ± 3	−49 ± 9
Dopamine	18 ± 2	10 ± 2*	−8 ± 2	−38 ± 8

Data are reported as mean ± SEM from *n* = 15 (Up‐Up and Down‐Down reported from *n* = 13 due to no sequences detected in *n* = 2).

*P < 0.05 versus Normoxia, ^†^ *P* < 0.05 versus Saline.

When examining the potential contribution of secondary outcome variables on changes in baroreflex sensitivity, we found the relative (%) fall in scBRS (Low‐Frequency Transfer Function) with hypoxia correlated with the change (%) in heart rate (*R* = −0.51, *P* < 0.01), tidal volume (*R* = −0.49, *P* < 0.01), and respiratory rate (*R* = 0.36, *P* = 0.05). The relative (%) fall in scBRS (Up‐Up Sequences) with hypoxia was also correlated with the change (%) in heart rate (*R* = −0.75, *P* < 0.01) and minute ventilation (*R* = −0.33, *P* = 0.10). The relative (%) fall in scBRS (Down‐Down Sequences) with hypoxia was correlated with the change (%) in heart rate (*R* = −0.70, *P* < 0.01), respiratory rate (*R* = −0.33, *P* = 0.10), and minute ventilation (*R* = −0.33, *P* = 0.10).

## Discussion

Novel findings indicate that the carotid chemoreceptors contribute to the fall in scBRS during acute hypoxia, such that decreasing activity of the carotid chemoreceptors with low‐dose dopamine significantly attenuates the reduction in scBRS during hypoxia; interestingly, this effect may be isolated to rising, versus falling, blood pressures. Given observed relationships between the fall in baroreflex sensitivity and respiratory parameters, our data also provide additional evidence of an interaction between the carotid chemoreceptors, pulmonary stretch receptors, and arterial baroreceptors in the observed responses. These results may have important implications for impairments in baroreflex function common in disease states of acute and/or chronic hypoxemia, as well as the experimental use of dopamine to assess such changes.

### Hypoxia and cardiac baroreflex sensitivity

Roche et al. (2002) have shown that when healthy adults are exposed to acute (~15 min) hypoxia (~80% S_p_O_2_), spontaneous cardiac baroreflex sensitivity is significantly reduced. Results from this study strengthen these findings and those of others (Heistad et al. 1971; Sagawa et al. 1997; Steinback et al. 2009). Specifically, we observed a significant (~50%) reduction in scBRS during hypoxia in the young, healthy adults studied. Although Roche and colleagues speculated that the changes in baroreflex sensitivity with hypoxia were the result of carotid chemoreceptor activation (Roche et al. 2002), the role of the carotid chemoreceptors in the observed response was not directly examined. However, data from populations with exaggerated carotid chemoreceptor activity (i.e., heart failure) support this idea, given impairments in cardiac baroreflex sensitivity are commonly observed. Furthermore, decreasing activity of the carotid chemoreceptors can significantly improve scBRS in such conditions (Ponikowski et al. 1999; Del Rio et al. 2013; Niewinski et al. 2013). Although these data lend support to our hypothesis, the specific contribution of the carotid chemoreceptors to changes in baroreflex sensitivity during acute hypoxia in healthy humans had not been directly investigated.

### Dopamine as an experimental tool

To examine the contribution of the carotid body chemoreceptors, subjects were exposed in a blinded, cross‐over design to an individual low‐dose of dopamine, shown to result in a significant reduction in the hypoxic ventilatory response (a measure of carotid body chemosensitivity to hypoxia, Fig. 1) (Limberg et al. 2016). Dopamine is an endogenous catecholamine known to act peripherally on both dopaminergic (D_1_ and D_2_) and adrenergic (*α* and *β*) receptors. D_2_ receptors play a main role in the depressant effect of dopamine, including reduced neurotransmitter release from type 1 glomus cells of the carotid body, followed by decreased neural output and carotid sinus drive (Black et al. 1972; Sampson 1972; Llados and Zapata 1978a; Bisgard et al. 1980; Lahiri and Nishino 1980; Ide et al. 1995; Nurse 2014). Importantly, low doses (<5 *μ*g·kg^−1^·min^−1^) of dopamine – similar to that used in this study – are thought to primarily target dopaminergic (D_1_ and D_2_) receptors (Black et al. 1972; Sampson 1972; Llados and Zapata 1978b; Bisgard et al. 1980; Lahiri and Nishino 1980; Ide et al. 1995; Ciarka et al. 2007), and only in higher doses, dopamine stimulates *α*‐ and *β*‐adrenergic receptors [thus directly affecting hemodynamic variables (Horwitz et al. 1962; Welsh et al. 1978; Lollgen and Drexler 1990; Ciarka et al. 2007)]. Furthermore, at low doses, dopamine is thought to bind mostly to D_2_ receptors as a result of higher affinity compared to D_1_ receptors (Lehmann et al. 1983). In this way, intravenous infusion of dopamine in low doses has been used in many studies in humans to acutely depress peripheral chemosensitivity to hypoxia (Welsh et al. 1978; Bainbridge and Heistad 1980; Ward and Bellville 1982, 1983; Boetger and Ward 1986; Sabol and Ward 1987; Bascom et al. 1991; Henson et al. 1992; Dahan et al. 1996; Ward et al. 2009; Stickland et al. 2011). To ensure that the dose of dopamine used would be sufficient to decrease carotid body afferent activity, while also limiting potential systemic cardiovascular effects which could independently affect baroreflex sensitivity, each participant completed a dopamine dosing visit and a significant fall in the ventilatory response to hypoxia, with minimal cardiovascular changes, was confirmed (Fig. 1; Limberg et al. 2016).

### Role of the peripheral chemoreceptors

When activity of the carotid chemoreceptors was decreased with low‐dose dopamine, the fall in scBRS during hypoxia was attenuated (Table 4, Fig. 2). However, this improvement was specific to the type of baroreflex sensitivity analysis applied (Low‐Frequency Transfer Function, Up‐Up Sequences), such that no improvements were observed in Down‐Down Sequences. Efferent control of heart rate is mediated by two effectors: vagal and sympathetic. It has been suggested that baroreflex sensitivity assessed by the transfer function method depends almost exclusively on vagal feedback gain to the heart (Pinna 2007; van de Vooren et al. 2007), although high sympathetic gain may also decrease baroreflex sensitivity measures in the low‐frequency range (Pinna 2007; van de Vooren et al. 2007). By assessing cardiac baroreflex sensitivity to both rising (Up‐Up) and falling (Down‐Down) blood pressures, we were able to further explore the effect of hypoxia on vagally mediated (parasympathetic system) and sympathetically mediated responses (La Rovere et al. 2008; Niewinski et al. 2014) in the presence/absence of active carotid chemoreceptors. Consistent with this, there is evidence of hysteresis in the baroreflex gain between rising and falling pressures (Studinger et al. 2007; La Rovere et al. 2008) and our results suggest the chemoreflex may influence each aspect differently.

We speculate that, although hypoxia contributes to a reduction in both vagal (Transfer Function, Up‐Up Sequences) and sympathetic (Down‐Down) arms of the baroreflex, desensitization of the carotid chemoreceptors results in a significant improvement in the parasympathetic (Transfer Function, Up‐Up Sequences) component of the autonomic nervous system only. These data suggest that the activation of the carotid chemoreceptors during hypoxia results in a primary attenuation of vagally mediated responses. However, it is important to note that although a rising sequence (Up‐Up) could be due to increased parasympathetic drive, parasympathetic withdrawal could also be the primary mechanism of a falling sequence (Down‐Down). Therefore, although our data provide important insight, our analysis does not allow for the strict identification of underlying mechanisms at this time.

### Potential mechanisms/location of interaction

The carotid chemoreceptors and arterial baroreceptors interact at a variety of levels. Although the location of this interaction was not directly examined in this study, it is reasonable to propose the change in baroreflex sensitivity may be attributed to an interaction between afferent activity of the carotid chemoreceptors and arterial baroreceptors at the level of the nucleus tractus solitarius (Mifflin et al. 1988; Mifflin 1993). For example, raising arterial blood pressure attenuates spontaneous and subsequent excitatory chemoreflex discharge from individual nucleus tractus solitarius neurons, suggesting convergent baroreflex afferents might directly inhibit, or disfacilitate, chemoreflex afferents.

Pure hypoxia is known to initiate reflex *decreases* in heart rate via carotid body mechanisms (Berk and Levy 1977; Kumar 2009). In contrast, the increase in heart rate commonly observed during hypoxia is a secondary response to hyperventilation (Hering–Breuer Reflex; Henson et al. 1992; Ursino and Magosso 2000; Kumar and Bin‐Jaliah 2007; Kumar 2009). For this reason, we also examined the relationship between the effect of dopamine infusion on tidal volume and subsequent changes in baroreflex sensitivity. Results show that those subjects with the greatest increase in tidal volume in response to hypoxia (i.e., greatest mechanoreceptor activation) also exhibited the greatest fall in scBRS. Given low‐dose dopamine is known to blunt the acute ventilatory response to hypoxia, it is reasonable to propose independent changes in lung stretch and/or changes in lung stretch secondary to attenuation of carotid chemoreceptor afferent activity contributed significantly to the observed change in steady‐state baroreflex sensitivity. Along these lines, Van De Borne et al. (2000) have shown an increase in tidal volume (i.e., increased lung stretch) of ~1 L significantly decreases arterial baroreflex sensitivity (~31% reduction). Results from this study show that significantly smaller changes in tidal volume (~200 mL) may have a similar effect on scBRS. With this in mind, future studies should consider more rigorous testing of the role of pulmonary stretch receptors and interrelationships with the carotid chemoreceptors on baroreflex sensitivity (e.g., having subjects control their tidal volume during the hypoxia exposure). Additional contributing factors may include independent effects of increased ventilation and/or hypoxia on the sinoatrial node or changes in central command (Van De Borne et al. 2000). These theories are supported by significant relationships between the change in baroreflex sensitivity and (1) heart rate and (2) respiratory rate.

The combined relationships between changes in baroreflex sensitivity and both tidal volume and respiratory rate may also suggest that inflation rate may be a critical factor in hypoxia‐mediated changes in baroreflex sensitivity. In this regard, Steinback et al. (2009) have also suggested that the rate of inspiration or ventilatory acceleration may be the stimulus for lung stretch receptor feedback mechanisms, and may also impact functioning of the cardiac baroreflex. For this reason, we also tested for potential relationships between baroreflex gain and inspiratory time; however, no significant relationships were observed.

### Experimental considerations

Although our data show that the carotid body chemoreceptors contribute to the reduction in scBRS during hypoxia, there are some important experimental considerations. First, a reduction in baroreflex sensitivity with hypoxia is not a universal finding (Cunningham et al. 1972; Eckberg et al. 1982; Knudtzon et al. 1991; Sagawa et al. 1997; Halliwill et al. 2003; Cooper et al. 2005; Fox et al. 2006) and results may be dependent upon the severity and/or length of hypoxic exposure studied. Second, the inspired oxygen levels necessary to achieve the same reduction in oxygen saturation (and P_a_O_2_, *n* = 6, data not shown) was less under dopamine conditions when compared to saline (Table 3). Although not a main focus of this study, dopamine has been shown previously to impair regional ventilation/perfusion matching in the lung [including increased pulmonary arteriovenous shunting (Huckauf et al. 1976; Shoemaker et al. 1989)], and although we do not observe any effect of dopamine on S_p_O_2_ during normoxia, our current data are supportive of this hypothesis. It is also possible that dopamine could result in peripheral vasoconstriction, thus affecting the results obtained from finger pulse oximetry. However, arterial blood gas data (S_a_O_2_) from a subset of subjects (*n* = 6) suggests this is an unlikely explanation. Third, this study focused on cardiac baroreflex sensitivity around the operating point of resting blood pressure and therefore results cannot be extrapolated out to sympathetic baroreflex sensitivity and/or more extreme swings in blood pressure. Further, we are unable to comment on the possibility of baroreflex resetting (Bristow et al. 1971; Knudtzon et al. 1991; Halliwill et al. 2003; Steinback et al. 2009). Fourth, the dose of dopamine used resulted in a significant reduction in the acute hypoxic ventilatory response (Fig. 1); however, after a ~4‐min transition to the desired ~85% S_p_O_2_, steady‐state respiratory rate, tidal volume, and minute ventilation during hypoxia were not different between saline and dopamine conditions (Table 3). We speculate that these differences are due to differences between ventilatory responses to short (seconds) versus longer term (minutes) hypoxic exposure (hypoxic‐ventilatory decline). It is known that during the initiation of hypoxia, ventilatory responses are quite dynamic and after an initial brisk increase in ventilation in response to acute hypoxia, levels typically decline. Our data are consistent with this notion (Table 3). Importantly, changes in carotid chemoreceptor discharge are thought to be responsible for the early increase in ventilation, whereas the hypoxic ventilatory decline is more likely centrally modulated (Georgopoulos et al. 1989). Lastly, hypoxic conditions were poikilocapnic and changes in P_a_CO_2_ are known to alter chemoreceptor activity. Importantly, reductions in end‐tidal CO_2_ (*n* = 15, Table 3) and/or P_a_CO_2_ (*n* = 6, data not shown) were not different between experimental conditions (saline, dopamine), thereby limiting any effect of changes in CO_2_ on main findings.

### Clinical perspective and conclusion

The carotid body chemoreceptors have recently been identified as a potential therapeutic target for sympathetically mediated conditions, such as heart failure and hypertension (Paton et al. 2013a,b). These patient populations often exhibit exaggerated carotid body chemosensitivity and impaired baroreflex sensitivity that can be improved with chemoreceptor‐targeted therapies. In this way, we also observed reductions in baroreflex sensitivity during acute chemoreceptor activation with hypoxia that improved when activity of the chemoreceptors was reduced with low‐dose dopamine. In heart failure, improvements in autonomic and cardiac function after carotid body desensitization/resection often occur after initial improvements in ventilatory parameters (Ponikowski et al. 1999; Marcus et al. 2014). The present results further suggest that changes in tidal volume may play a role in observed improvements in baroreflex sensitivity during hypoxia. Although only healthy individuals were studied in the present investigation, the combined data further support a body of literature showing a strong influence of the carotid body chemoreceptors on cardiac baroreflex sensitivity. These findings have relevance to our understanding of impairments in baroreflex function and the progression of hypertension and cardiovascular complications observed under conditions of acute and/or chronic carotid body activation with hypoxia, as well as the experimental use of dopamine to assess such changes.

## Conflict of Interests

There are no competing interests and no relevant conflicts of interest.
